# Description of a Pharmacy COVID Champion Service in South East London to Reduce Vaccine Hesitancy

**DOI:** 10.3390/pharmacy10060143

**Published:** 2022-10-28

**Authors:** Ricarda Micallef, Raj Matharu, Abigail Barry, Vanessa Burgess

**Affiliations:** 1 Department of Pharmacy, Penrhyn Road Campus, Kingston University London, London KT1 2EE, UK; 2 Bromley, Bexley and Greenwich and Lambeth, Southwark and Lewisham Local Pharmaceutical Committees, London BR4 0PX, UK; 3 NHS South East London Integrated Care System, London SE1 2TZ, UK

**Keywords:** pharmacist, COVID, vaccination, hesitance

## Abstract

In the United Kingdom, COVID vaccinations were rolled out from December 2020. In July 2021 in South East London there were areas of high COVID prevalence and low vaccination uptake. Therefore, a COVID champion service was launched in community pharmacies enabling pharmacy teams to have conversations with patients regarding the vaccination programme and their concerns and signposting as needed. The aim of the project was to evaluate the impact of the service on COVID vaccination uptake and perceptions of pharmacy teams. Quantitative data was received from pharmacy interaction records plus a pharmacy survey. In addition, qualitative data was received through interviews with 12 pharmacists involved in the service, which was analysed using content analysis, along with interviews with commissioner representatives. Between July and October 2021, 8539 conversations took place. From these 6094 patients agreed to vaccination, with 2019 initially hesitant patients converted. Lack of understanding, risk of blood clots and cultural concerns were the largest areas of hesitance. Pharmacy teams were happy to support conversation and local working with knowledge and confidence. Engagement within the team was the biggest enabler, with pressure to deliver other services being the biggest barrier. These results show the value of community pharmacy teams, having conversations with the public, addressing concerns where applicable, and signposting to the appropriate sites so patients are supported to achieve maximum health outcomes.

## 1. Introduction

COVID vaccinations started to be rolled out in December 2020 across the United Kingdom (UK) [1]. Initially the most vulnerable were offered vaccination, starting with all residents in care homes, followed by over 80 years of age, then front line healthcare workers, individuals over 75 years of age, then over 70 years of age or clinically extremely vulnerable. In April 2021 additional age groups were offered a vaccination, working down in 5-year age group steps. From June 2021 all those over 18 years of age were eligible. In South East London (SEL) the Clinical Commissioning Group (CCG), overseen by ‘=’ Our Healthier South East London’ [2], working with the Local Pharmaceutical Committees (LPCs) identified a need to continue to engage the local community in the vaccination programme to increase uptake. CCGs are clinically led statutory bodies who commission most of the hospital and community National Health Service (NHS) services in the local areas for which they are responsible [3]. LPCs are the focus of all community pharmacists and is an independent and representative group, negotiating local services, and support local community pharmacy provision [4]. SEL consists of six boroughs: Lambeth, Southwark, Lewisham, Bexley, Bromley, and Greenwich, with a population of approximately 1.8 million. Although these Boroughs, for pharmacy services, are overseen by an umbrella CCG there are local Borough teams who can target specific localised services. Across SEL in summer 2021, there were pockets of areas that had a high prevalence of COVID positive patients but also had low uptake of vaccination. Some of the target areas have high levels of ethnic minorities, who are known to be more vaccine hesitant, or have more access issues to receiving the vaccination [5,6]. Studies show that vaccine coverage was lowest for those who described their ethnicity as African, followed by Bangladeshi and those of Caribbean ethnicity [7,8]. There are known reasons for vaccine hesitancy [5,6], and the COVID champion service concept wanted to address some of the hesitance by open conversations, and supporting individuals to be signposted to their local vaccination centre (LVS) or to support booking of an appointment using the national booking system (NBS). Signposting to local centres gave information about locations, booking and access, to increase confidence and uptake. The importance of vaccine public information that promotes prosocial benefits has previously been noted [9]. Addressing areas of concern and lack of understanding, along with increasing awareness and answering questions to address hesitance was seen as a positive approach. The government recognises the need for ongoing community engagement, tailored communication, practical support, and training for all healthcare staff to have trusted conversations [10].

In a literature review on COVID-19 vaccination hesitancy [11] the biggest reason for hesitance was being against vaccines in general then safety concerns, and lack of trust. Multiple reports have shown the value of the COVID vaccination, reducing hospital admissions, and preventing severe disease. Although numbers have changed with increased vaccination One report [12] from October 2021 showed that emergency hospital admissions were 51% lower in vaccinated individuals 21–27 days after vaccination and 76.5% lower after 35–41 days. Positive COVID-19 tests were 55.2% lower for vaccinated individuals after 21–27 days and 70.1% lower after 35–41 days. When looking at hospitalisation, a paper [13] estimated the average cost of a hospital stay for a patient with COVID-19 to be £4847. From government data [14] it is seen that for London as a whole, until the end of September 2021 there had been 25553 hospital admissions for COVID-19 with the average hospitalisation rate for the whole of London until the end of September being 4.97 per 100,000 people [15].

Community Pharmacies are uniquely placed to support an increase in vaccination coverage through a “every contact counts” approach. There are 324 community pharmacies in SEL with 89% of the population living within a 20 min walk of a community pharmacy [16]. Community pharmacy in South London, including SEL plus the pharmacies in South West London, has a wide network of health champions [17], who can have conversations with patients to support health outcomes.

Pharmacies stayed open throughout the pandemic, and the positive role of pharmacies is commended by the local community, such as in a report by Healthwatch in Lewisham that stated most people experienced ‘organised and professionally managed’ pharmacy services [18]. Community pharmacists have multiple roles to play in supporting the wider community and healthcare team through activities linked to prevention, preparedness, response and recovery [19,20]. Community wellness outreach also allows discussion on how to self-care, improve wellness, explain the pharmacist role in helping people look after their own health, and services that pharmacists offer including vaccinations [21]. Community education can support the dissemination of messages in an environment where individuals feel safe and valued, and help the community understand the role of the pharmacy team, to reduce health inequalities [21].

The COVID Champion service was launched in July 2021 with an initial end date of September 2021. The COVID champion service aimed to increase vaccination coverage across SEL by capitalising on the position of community pharmacy. The Vaccination Champions worked in line with the principles of “Making Every Contact Count.”

The aim of the project was to evaluate the impact of the COVID champion service in South East London on COVID vaccination uptake to identify the patients who were targeted, identifying issues of hesitance, along with ascertaining perceptions from pharmacies involved in the service, such as barriers, enablers and ongoing support requirements.

## 2. Materials and Methods

### 2.1. Service Overview

The principles of the service involved brief intervention conversations, opportunistically identifying patients and residents who had not had their vaccine, especially those who had been eligible for longer periods of time, and then discussing the vaccination programme and, with consent, booking the patient for a vaccination or signposting them to their local walk-in centre.

Pharmacies were paid £8 sterling per intervention conversation that was logged onto PharmOutcomes, a pharmacy data collection system, where hesitancy was documented. PharmOutcomes is a system used nationally by community clinicians for recording interventions to allow commissioners to audit and manage service delivery. The expectation was that pharmacies would have between 25–85 conversations weekly. Pharmacy teams were given information websites to increase their knowledge of vaccinations and services locally, to support conversations.

The service outline in community pharmacy incentivised community pharmacy champions to have either a brief advice, a timeframe of up to 3 min or brief intervention, which could be up to 30 min then record the outcomes. The service was about opportunistically identifying patients and residents who came into the community pharmacy, primarily targeting over the age of 40 who have not had their vaccination, discussing the vaccine programme with these patients to increase vaccine confidence. After discussion, with patient consent, patients that agreed to vaccination would be booked onto the UK National Booking System or guided towards a local walk-in centre. Patients could also be immediately offered a vaccine as a walk-in if the community pharmacy already offered a local vaccination site service.

In addition to the COVID champion service, engagement with local communities was enhanced through Community Pharmacy Wellness Dialogues. Overall, nine sessions were completed, led and coordinated by Making Connections Happen. Community Pharmacy Wellness Dialogues were developed to provide outreach support through both online and face-to-face engagement targeted at the African and Caribbean population groups across multiple settings including churches, social organisations and business groups. They aimed to raise the profile of community pharmacists, improve vaccine uptake and vaccine confidence and raise community pharmacy and public health awareness for the needs of the African and Caribbean population within local areas. Using community pharmacy colleagues was also a key part of the programme as ‘trusted messengers.’

### 2.2. Study Design

A retrospective cross-sectional study was completed, that examined the implementation of the programme at participating pharmacies. Quantitative data was collected and reported by pharmacies after conversations through the pharmacy collection tool PharmOutcomes. Data was collected regarding the individuals demographic detail (age, gender and ethnicity, as identified by the United Kingdom government [22], along with hesitance, and their reason for hesitance, along with outcome after the conversation. Every pharmacy also received a pharmacy survey consisting of 15 questions, asking about experiences of the service, such as motivations to be involved, information about the service delivery, enablers and barriers for delivery, and the knowledge and confidence of the team. This was online using JISC online surveys. Finally, qualitative data from pharmacists, along with CCG, LPC and commissioner representatives, using semi-structured, one-on-one interviews. Interviews were completed to understand perceptions and experiences in more detail. An interview proforma consisting of 13 semi-structured questions was designed for pharmacists aiming to extrapolate the findings from the survey. For CCG, LPC and commissioner representatives the interview contained just five questions. The interview schedules were adapted from services used for previously completed evaluations therefore received no additional face validation or piloting.

### 2.3. Participants: Sampling and Recruitment

Human participants were involved in data collection through completion of an online survey and semi-structured telephone interviews. The lead author completed all the interviews. A sample of 20 participating pharmacies were approached to take part in a semi-structured interview, to gain some case studies and get more in-depth detail. Initially a sample of pharmacies was selected for interview after reviewing the number of conversations in the pharmacy, and identified the five who completed the most conversations, the five who completed the least and the remaining ten being middle for number of completed conversations. After two emails no pharmacy had come forward. Thereafter, through completion of the survey pharmacies were asked to provide contact details (email address/phone number) if they were willing to participate in a follow up interview. All those who left contact details were reached out to. Reminders were sent to increase responses, but limited numbers came forward. All those who responded were interviewed. All pharmacies in SEL were contacted with the online survey link via email distributed on behalf of the lead author by the LPC. The CCG, LPC and commissioner representatives were contacted directly for interview. CCG, LPC and commissioner representatives were contacted to understand their organisations perspectives on the service, and to gain feedback from those they represent. Participants, when contacted, were made aware of the survey and interview being part of the evaluation work commissioned to evaluate the service. They were also made aware of her credentials and practise as a pharmacist.

### 2.4. Data Collection

Individuals who agreed to participate in an interview were emailed an information sheet, outlining the study aims and objectives and the background of the researcher, including the right to withdraw from the study, and a consent form, which they were asked to read, sign and return, prior to the agreed interview time. The researcher had no prior relationship with the pharmacists. All interviews occurred over the telephone for convenience. Verbal consent was obtained for recording. Interviews lasted between 7 to 16 min. All interviews were voice recorded and transcribed verbatim prior to deletion. No other notes were made during the interviews. All those who initially agreed to be interviewed completed an interview. One female member of the research team (RM), who has five years prior experience of qualitative research from PhD study completed all the interviews and completed the transcriptions. No other individuals besides the researcher and participant were present during all interviews. A copy of the interview schedules can be found in Appendix A and Appendix B. A copy of the Consolidated Criteria for reporting Qualitative Studies (COREQ) checklist to ensure integrity in design and analysis of qualitative data, can be found in Appendix C.

Collection of data in pharmacies about the number of conversations they had was collected between July and September 2021. Survey data was collected in October and November 2021 and interviews were carried out between November and December 2021.

### 2.5. Data Analysis

Pharmacy data was analysed from Microsoft Excel and reviewed by Borough and demographics or age, gender and ethnicity, to highlight areas where greater focus as needed. Analysis reviewed hesitancy and outcome by demographic, with descriptive statistics being used as necessary, where significance is *p* ≤ 0.05. Survey data was downloaded to Microsoft Excel for analysis. Overall data was reviewed, using weighted means, where appropriate. Inductive content analysis was used to combine data gathered through interview to give a general statement. Working with qualitative data, content analysis allows the integrity of the narrative to be maintained [23]. The questions were used as codes. This was completed by one member of the research team (RM), as the individual with experience of analysis, with all transcripts made available for the rest of the research team. Analysis was completed manually. Transcripts were read to ensure there were no transcription errors, and then read again to enable immersion. The codes under the key objectives are given in italics.The project gained ethical approval from Kingston University (2910).

## 3. Results

### 3.1. Pharmacy Data

Only data from pharmacies up until the end of September 2021 was analysed. In that time a total of 8539 intervention conversations were recorded. Per pharmacy it ranged from 1–603 conversations. In total, 123 pharmacies were involved in the service. Pharmacies represented by the Company Chemists Association, which represents pharmacies from large chains, were not involved in the service due to contractual arrangements.

By Borough, Lewisham completed the most conversations completing a quarter of the conversations (*n* = 2173/8539). Although Bromley, Lambeth and Southwark have the highest populations, these boroughs saw the fewest conversations.

When looking at conversations by demographic, the majority of those spoken to were in the age range 41–50, echoing the service detail to focus on those over 40. Overall, 60% (*n* = 5118/8539) conversations were had with those over the age of 40. Noting that ethnicity was self-defined by those who participated in the service, by numbers White British were the most represented group. When comparing white versus non-white categories overall, conversations with those who defined themselves as white were 43.4% of the total (*n* = 3700/8539), with non-white being 50.8% (*n* = 4340/8539) and prefer not to say at 5.8% (*n* = 499/8539). By gender 55% of those who participated were female. Full demographic detail can be seen in Table 1.

From the conversations that have taken place just over half (52%, *n* = 4464/8539) were hesitant. In terms of hesitation for the 4464 patients, lack of understanding was the largest reason with 31.9% (*n* = 1425/4464) stating this reason, followed by concerns with blood clots (26.1%, *n* = 1164/4464) and cultural/family concerns (12.2%, *n* = 546/4464). Hesitance by gender can be seen in Table 2, hesitance by age in Table 3 and hesitance by ethnicity in Table 4 and Table 5.

As seen in Table 2, Table 3, Table 4 and Table 5, the top reason differs by demographic, showing different groups have different concerns. When looking at the top 5 reasons for hesitation by gender the transgender group were most worried about blood clots. Males were most likely to have a lack of understanding and cultural and family concerns. Females were most likely to be worried about fertility, pregnancy, and breastfeeding. When looking at hesitation by age half of those over 90 were hesitant due to lack of understanding. Worry about fertility, pregnancy and breastfeeding was statistically more likely in younger groups (*p* = 0.0031). By gender no significant difference was seen between groups (*p* = 0.3358), but differences between ethnicities was significant (*p* = 0.0000). Hesitation by ethnicity shows that Asian or Asian British-Pakistani had high cultural/family concerns (36.7%, *n* = 80/218), but were the lowest for lack of understanding (17.4%, *n* = 38/218). Arab had the highest hesitation due to cultural/family concerns (37.5%, *n* = 18/48) but were the lowest for concerns about blood clots (10.4%, *n* = 5/48). Lack of understanding was highest in Asian or Asian British-Chinese (41.4%, *n* = 48/116) and other ethnic group (41.7%, *n* = 25/60). Concern regarding blood clots was highest for those from Black or Black British-other black background (45.6%, n = 26/57). Table 6 outlines the overall hesitance and agreement to vaccination data.

From all conversations, a total of 6094/8539 patients (71.4%) agreed to vaccination, therefore 1630 hesitant patients were converted. Therefore, overall conversion was 19.1% of conversations. When looking at conversion to vaccination from initial hesitation by demographic, younger age groups were initially more hesitant, but those aged 18–20 were most likely to agree to vaccination after a conversation. Other than those aged 18–20, those over 60 saw the highest agreement rates, a significant difference (*p* = 0.0011). Those from ethnic minority group were initially most hesitant but overall agreement did not differ significantly by ethnicity (*p* = 0.5176) or gender (*p* = 0.6907) after a conversation There were limited differences seen in different genders.

Of the 6094 patients who agreed to vaccination the majority of these were signposted to a LVS or the NBS. A fifth (20.1%) were also vaccinated at the pharmacy, showing the value of having vaccination centres on site at the pharmacy. All the outcomes for those who agreed were positive, with signposting or bookings being made.

From the 8539 conversations 28.6% (*n* = 2445) were listed as not agreeing to vaccination. However, when looking at outcomes for these patients, even when patients did not initially agree to vaccination, a minority were also signposted to a LVS or the NBS. Overall outcomes can be seen in Table 7, with Table 8 outlining the top outcomes by demographic.

Looking at the numbers, those who agreed to vaccination are far higher than those who did not agree. Looking at the overall top 5 outcomes by demographic, by gender these are similar. When looking at outcome by age, older patients were more likely to be signposted to a LVS, with younger patients more likely to be signposted to the NBS, although the difference is not significant. Younger patients were also likely to be vaccinated in the pharmacy. Almost a fifth of those over the age of 90 (17.6%) did not agree to vaccination. By ethnicity white British were most likely to be signposted to a LVS (33.6%), followed by Asian or Asian British-Indian at 27.8%. The Asian or Asian British-Indian group were the lowest for not agreeing to vaccination (3.4%). Other ethnic group were the most likely to be vaccinated in the pharmacy (37.8%) followed by white other at 32.4%. Almost half (45.7%) of Asian or Asian British-other Asian background were signposted to the NBS. More information was given most to those of black or black British-African (11.7%) and Caribbean (14.3). However, no significant differences were seen.

### 3.2. Pharmacy Questionnaire

In total 54 responses were received (42% response rate). Most pharmacies stated that they had completed up to 50 conversations on the service (*n* = 26; 48%), with 4 responders (7.4%) completing over 500, echoing the pharmacy data showing pharmacies completed between 1–603 conversations.

Of the 46 pharmacies answering that question, stated that there were between 1 (*n* = 2) to 9 members of staff (*n* = 3) involved in the service in the pharmacy, with all roles included, from counter assistant through to Pharmacist. The mean number of pharmacy staff was 4. Almost all conversations took less than 10 min (*n* = 48/53, 90.5%), with the most taking less than 5 (*n* = 27/53, 50.9%).

#### 3.2.1. Motivators for Signing Up to the Service

When asked why pharmacies had signed up to the service most responses were to support vaccination uptake and support local health needs and positive outcomes for patients. There was understanding of the outcomes of the service seen. The main words seen in the comments were vaccination, COVID, patients, help, promote, encourage showing positive motivations for signing up. When asked how the pharmacy promoted the service to patients, conversations with patients who had come in for an over the counter (OTC) purchase or to pick up a prescription were the most common reasons cited.

#### 3.2.2. Thoughts about the Service

When asked to rank aspects of the service, with 1 being the lowest and 10 being the highest, mean scores were calculated, and these can be seen in Table 9.

When asked to rank aspects of the service, whilst all scores were positive, more promotion material was requested, and this was echoed in interview responses.

#### 3.2.3. Enablers for the Service

When asked about enablers for the service, an engaged team (*n* = 44/54, 83%) and all the team being involved (*n* = 36, 67.9%) were seen as the biggest enablers. The incentive of payment was also seen as an enabler (*n* = 29/54, 54.7%).

#### 3.2.4. Barriers for the Service

When asked the tick the barriers for the service, where multiple options could be selected, pressure to deliver other services (*n* = 35/54, 64.8%), patients not willing to share their details (*n* = 29/54, 53.7%) or limited patients to ask (*n*−27/54, 50%) were seen as the top 3 barriers.

#### 3.2.5. Ongoing Support Requirements

Pharmacists were asked for suggestions for future roll outs linking to training, resources, promotion and team engagement. For resources, leaflets to give to patients were requested by almost all who responded. Internet based resources for signposting could also be considered. Posters were the most requested promotion tool, along with leaflets, and online campaigns supported locally and nationally. Team engagement varied by pharmacy, but linking to the comments received for training, promotion and resources, all the team should be engaged, using easy to understand resources and have the tools to be able to record conversations appropriately.

### 3.3. Interviews with Pharmacists

In total, 12 interviews took place with pharmacists involved in the COVID champion service. The responses echo what has been seen from the questionnaire.

#### 3.3.1. Motivators for Signing Up to the Service

Pharmacists are happy to offer the service as they believe they are well placed to support the effort to vaccinate the local population. Multiple pharmacists commented that they were already having conversations with patients, so this service was an opportunity to formalise the discussions.

#### 3.3.2. Thoughts about the Service

Although positive, some pharmacists did comment that some patients had been asked multiple times about their vaccination status, as it is hard to remember who you have asked. In addition, pharmacists stated that, although, the service was hugely positive, many of their patients had now been spoken to, showing the service had fulfilled its intended purpose. Most pharmacists stated that they asked regular patients or had the conversation with everyone who came into the pharmacy.

Being able to convert patients, who did not initially believe they were able to have the vaccination, was identified. Pharmacists noted that most patients were very happy to have the conversation. Reluctance to have the conversation came from information that needed to be given, such as postcode. In addition, there are some patients that have already made their decision and will not be converted.

Hesitancy echoed that seen in the data from pharmacies, with most of the hesitance they had encountered being around lack of understanding, cultural reasons or concerns around blood clots or fertility. Another key issue identified was patients who did not know how to book appointments, and the pharmacies were able to support this.

#### 3.3.3. Enablers for the Service

The pharmacists interviewed identified the positive aspects of pharmacists being involved in vaccination conversations, and in vaccination services, and that patients continue to see pharmacists as a source of information and support in the community. It is also seen that patients are seeing the real value of pharmacists, beyond dispensing.

Pharmacists also feel a valued member of the local community and healthcare team. In addition, this service has allowed all the team to be involved in conversations. All pharmacists identified that all their pharmacy team were engaged in the service and felt confident to have conversations.

Other enablers not captured in the pharmacy survey include a diverse team to communicate with patients. Linking to other services, such as lateral flow testing, or being a COVID vaccination site was also a large positive to enable the service to be successful. Where the pharmacy was not a vaccination site, local relationships facilitated signposting and uptake.

#### 3.3.4. Barriers for the Service

In terms of barriers, most of those seen were noted in the pharmacy questionnaire, such as data requirements, and time. Patients not willing to have a conversation or being approached multiple times was also noted.

#### 3.3.5. Ongoing Support Requirements

To support the service going forward, as seen in the suggestions from the pharmacy questionnaire more training and promotional material would be useful.

### 3.4. Greater Collaborations for Moving Forward

The overall response from participants at outreach events was positive and much appreciated that a Community Pharmacists was willing to attend their meetings and provide a comprehensive overview of the services they provided relating to wider health and wellness with a general response that there was a lack of awareness of the full range of services being offered by Community Pharmacy although they are trusted members of their local communities and neighbourhoods.

During their interviews, both the commissioner and LPC recognise the need to work together going forward, and how community pharmacy can be utilised most effectively, and work within an integrated care setting. The legacy of the service should be that the commissioner (CCG) and provider (LPC) need infrastructures in place to have all paperwork and agreements in place so if a new service is required it can be turned around quickly and at pace. There is a continued need to work at pace with increased relationships to deliver services that will impact on public health.

## 4. Discussion

These results show the value of community pharmacy, having conversations with the public, addressing concerns about vaccination hesitance, and then signposted, where applicable, to the appropriate sites so patients are supported to achieve the maximum health outcomes possible.

From interviews and the pharmacy survey, pharmacists are confident and knowledgeable to have conversations, as well as accessible to the public, and well placed at the centre of their communities. The results of this study echo previous studies [24,25] that show value of community health champions in supporting patient conversations, with multiple pharmacy roles having importance. This study highlighted similar facilitators and barriers to service delivery as a previous paper, including access to information, time and competing priorities [24]. Whilst patient outcomes are positive, the need for continued training and the need for patient follow up is also seen in a previous paper on South London health champions [25]. Results were received from multiple sources, and all correlated, showing similar outcomes, enablers and barriers, plus positive feedback about the service and suggestions for future services. This service also highlights the positive outcomes from collaborations from across the healthcare system.

The key results show the positive interventions that were achieved during the COVID champion service, targeting key individuals who were supported with positive conversations, in a timely manner, and who were supported to be vaccinated. Hesitations were addressed and information given, to support public health outcomes. Conversations were linked to other lifestyle and COVID related services already run in the pharmacy. As all the pharmacy team were involved this supported whole team conversations.

The results from the pharmacy questionnaire showed that most conversations took less than 10 min, with many taking less than 5. Future services should review the length of conversations, and perhaps review a brief intervention conversation approach, followed by a longer conversation, if required, or multiple brief intervention conversations, depending on the service. However, it should be noted that a Cochrane Review in 2007 identified that longer brief interventions offered no significant benefit over shorter input [26]. A brief intervention would be classed as a conversation that lasts between 5–15 min [27]. To support having effective conversations there is evidence to support the use of motivational interviewing, that would support the change process [28].

The pharmacy PharmOutcome results show that a large number of the population were vaccine hesitant, but many of these were able to be converted after an appropriate conversation. Lack of understanding was seen to be the reason that the majority in this study were hesitant. This has also been seen previously where hesitant individuals had gaps in knowledge compared to accepting individuals [29]. Outreach conversations, focusing on local populations and local issues, may also support ongoing education, as seen in this study.

Whilst the results of this study from the pharmacy data showed that Bromley, Lambeth and Southwark saw the lowest number of conversions, these were the boroughs that had high vaccination uptake. Government data showed that up until 3rd October 2021 just over 2.3 million vaccinations had been delivered across SEL including boosters [30]. From the 6094 in this sample who agreed to vaccination, infection rates and hospitalisation would have been reduced, minimising overall burden on the NHS.

This study did not show any significant differences in hesitancy by gender or ethniticy, and this echos a previous study [9] where hesitancy was evenly spread across the population. This study showed that age affected conversion after conversations, with more patients aged 18–20 and over 60 years of age being converted. However, whilst demographic data was explored in our cohort, we did not look at other lifestyle factors that may also impact hospitalisation, such as smoking and weight [31].

The COVID-19 pandemic continued to build trust in pharmacies and build on being the ‘most trusted health care professional’ [32,33], which may also help to explain why this service through community pharmacies was so successful. It is seen that when it comes to vaccine uptake healthcare provider communications and behaviour strongly influence patient behaviour and uptake [34] therefore trust in the provider is essential to ensure a positive outcome.

There were some limitations to this study. There was no involvement of multiple pharmacies due to contractual issues. This has been identified as something to address through an integrated care system going forward. The initial service also did not require participating pharmacists to gather immediate patient feedback. In addition, only one individual coded the qualitative data.

As a result of the findings in this report some recommendations can be made for future public health services. Community pharmacy should continue to be used for community engagement and outreach, engaging with all the population, especially those hard to reach and more vulnerable groups, due to their involvement in local communities. Community pharmacy needs to be reimbursed for specific/defined services delivered in support of public health including outreach clinic services in a timely manner. Systems need to ensure communication channels are in place to gather learnings and expertise from all parties involved in a service. Investment is required to allow pharmacists to be relieved from their pharmacies to contribute to outreach work, supporting medicines and public health outcomes. All the pharmacy team should continue to be engaged in health promotion, and further lifestyle conversations, supporting overall health outcomes, such as other vaccinations, blood pressure or cholesterol testing.

## 5. Conclusions

The COVID champion service successfully enabled conversations in community pharmacy to support patients in overcoming hesitance towards the COVID vaccination, covering a wide range of demographics. Multiple pharmacists were already having conversations with their patients about vaccinations. Pharmacists are positive about the impact of the service, with the knowledge and confidence to have conversations. Payment to recognise the important role of the community pharmacist enables conversations, and more staff to be recruited. It also supports pharmacists to feel valued and acknowledged for the role they play, even if more conversations take place outside of the remit of a service. Barriers to be overcome for delivery of future services include time, and patients being willing to have the conversation.

The largest reason for hesitance identified was lack of understanding. However, conversion from hesitancy is possible when patients engage in a brief conversation. The service has seen almost 1 in 5 patients converted from hesitation to agreeing to the vaccination. The key metrics of the service showed that a large majority of patients were signposted to the NBS or a LVS, and a fifth were vaccinated in a community pharmacy, showing the value of the community pharmacy team in supporting health outcomes. Community pharmacies are central to local communities and have established local relationships to support signposting.

Pharmacists feel that patients trust them for advice and community pharmacists are central to localities, as trusted messengers, and part of the overall primary care team, providing services and health promotion and advice. Pharmacists have the knowledge and confidence to deliver services, along with their teams, and the public value this. Community pharmacists are accessible and have good relationships with their patients.

Overall, from patient outcomes and pharmacist experiences, the COVID champion service has been a positive service for community pharmacy, the local community and public health outcomes. Community pharmacy should continue to be funded, and receive ongoing training and development, to support health interventions to support patient and public health outcomes.

## Figures and Tables

**Table 1:** Demographic detail of individual conversations. Total *n* = 8539.

Borough (*n* = 8539)		Ethnicity (*n* = 8539)	
Bexley	*n* = 2030 (23.8%)	Arab	*n* = 88 (1.0%)
Bromley	*n* = 709 (8.3%)	Asian or Asian British-Chinese	*n* = 245 (2.9%)
Greenwich	*n* = 2126 (24.9%)	Asian or Asian British-Indian	*n* = 500 (5.9%)
Lambeth	*n* = 798 (9.3%)	Asian or Asian British-Other Asian background	*n* = 138 (1.6%)
Lewisham	*n* = 2173 (25.4)	Asian or Asian British-Pakistani	*n* = 333 (3.9%)
Southwark	*n* = 703 (8.2%)	Black or Black British-African	*n* = 1477 (16.9%)
**Age (*n* = 8539)**		Black or Black British-Caribbean	*n* = 692 (8.1%)
18–20	*n* = 591 (6.9%)	Black or Black British-Other Black background	*n* = 95 (1.1%)
21–30	*n* = 1522 (17.8%)	Mixed-Other mixed background	*n* = 65 (0.8%)
31–40	*n* = 1308 (15.3%)	Mixed-White & Asian	*n* = 157 (1.8%)
41–50	*n* = 2503 (29.3%)	Mixed-White & Black African	*n* = 226 (2.6%)
51–60	*n* = 1418 (16.6%)	Mixed-White & Black Caribbean	*n* = 235 (2.8%)
61–70	*n* = 709 (8.3%)	Other ethnic group	*n* = 119 (1.4%)
71–80	*n* = 346 (4.1%)	Prefer not to say	*n* = 499 (5.8%)
81–90	*n* = 125 (1.5%)	White-British	*n* = 2854 (33.4%)
91–100	*n* = 17 (0.2%)	White-Gypsy or Irish traveller	*n* = 87 (1.0%)
**Gender (*n* = 8539)**		White-Irish	*n* = 228 (2.7%)
Female	*n* = 4655 (54.5%)	White-other	*n* = 531 (6.2%)
Male	*n* = 3862 (45.2%)		
Transgender	*n* = 22 (0.3%)		

**Table 2:** Reasons for hesitance by gender.

Reason for Hesitation	Overall (*n* = 4464)	Female (*n* = 2466)	Male (*n* = 676)	Trans-Gender (*n* = 6)
Lack of understanding	*n* = 1425 (31.9%)	*n* = 676 (27.4%)	*n* = 748 (37.6%)	*n* = 1 (17%)
Concerns with blood clots	*n* = 1164 (26.1%)	*n* = 621 (25.2%)	*n* = 538 (27.0%)	*n* = 6 (83%)
Cultural/family concerns	*n* = 546 (12.2%)	*n* = 262 (10.6%)	*n* = 284 (14.3%)	*n* = 0 (0%)
Risk of fertility	*n* = 280 (6.3%)	*n* = 217 (8.8%)	*n* = 63 (3.2%)	*n* = 0 (0%)
Pregnancy/breastfeeding	*n* = 269 (6.0%)	*n* = 256 (10.4%)	*n* = 13 (0.7%)	*n* = 0 (0%)
Does not want it-no additional reason	*n* = 153 (3.4%)	*n* = 92 (3.7%)	*n* = 61 (3.1%)	*n* = 0 (0%)
Not comfortable or confident yet	*n* = 137 (3.1%)	*n* = 81 (3.3%)	*n* = 56 (2.8%)	*n* = 0 (0%)
Side effects	*n* = 87 (1.9%)	*n* = 36 (1.5%)	*n* = 51 (2.6%)	*n* = 0 (0%)
Not enough evidence	*n* = 78 (1.7%)	*n* = 44 (1.8%)	*n* = 34 (1.7%)	*n* = 0 (0%)
Health issues	*n* = 52 (1.2%)	*n* = 28 (1.1%)	*n* = 24 (1.2%)	*n* = 0 (0%)
Does not believe it is needed	*n* = 44 (1.0%)	*n* = 18 (0.7%)	*n* = 26 (1.3%)	*n* = 0 (0%)
Does not trust it	*n* = 33 (0.7%)	*n* = 17 (0.7%)	*n* = 16 (0.8%)	*n* = 0 (0%)
Anti vaccine	*n* = 25 (0.6%)	*n* = 13 (0.5%)	*n* = 12 (0.6%)	*n* = 0 (0%)
Not had time	*n* = 24 (0.5%)	*n* = 9 (0.4%)	*n* = 15 (0.8%)	*n* = 0 (0%)
Scared	*n* = 24 (0.5%)	*n* = 19 (0.8%)	*n* = 5 (0.3%)	*n* = 0 (0%)
Needle phobia	*n* = 20 (0.4%)	*n* = 14 (0.6%)	*n* = 6 (0.3%)	*n* = 0 (0%)
No reason given	*n* = 18 (0.4%)	*n* = 13 (0.5%)	*n* = 5 (0.3%)	*n* = 0 (0%)
Allergies	*n* = 17 (0.4%)	*n* = 12 (0.5%)	*n* = 5 (0.3%)	*n* = 0 (0%)
Could not make an appointment	*n* = 17 (0.4%)	*n* = 8 (0.3%)	*n* = 9 (0.5%)	*n* = 0 (0%)
Personal reasons	*n* = 14 (0.3%)	*n* = 9 (0.4%)	*n* = 5 (0.3%)	*n* = 0 (0%)
Adverse reaction to previous vaccine	*n* = 12 (0.3%)	*n* = 8 (0.3%)	*n* = 4 (0.2%)	*n* = 0 (0%)
Had COVID	*n* = 8 (0.2%)	*n* = 5 (0.2%)	*n* = 3 (0.2%)	*n* = 0 (0%)
Does not want to be told what to do	*n* = 7 (0.2%)	*n* = 4 (0.2%)	*n* = 3 (0.2%)	*n* = 0 (0%)
Does not believe COVID exists	*n* = 5 (0.1%)	*n* = 1 (0.0%)	*n* = 4 (0.2%)	*n* = 0 (0%)
Wants specific brand of vaccine	*n* = 5 (0.1%)	*n* = 3 (0.1%)	*n* = 2 (0.1%)	*n* = 0 (0%)

**Table 3:** Reasons for hesitance by age.

Reason for Hesitation	Overall (*n* = 4464)	Aged 18–20 (*n* = 234)	Aged 21–30 (*n* = 901)	Aged 31–40 (*n* = 747)	Aged 41–50 (*n* = 1442)	Aged 51–60 (*n* = 736)	Aged 61–70 (*n* = 282)	Aged 71–80 (*n* = 93)	Aged 81–90 (*n* = 25)	Aged 91–100 (*n* = 4)
Lack of understanding	*n* = 1425 (31.9%)	*n* = 83 (35.5%)	*n* = 296 (32.9%)	*n* = 201 (26.9%)	*n* = 461 (32.0%)	*n* = 246 (33.4%)	*n* = 99 (35.1%)	*n* = 0 (0.0%)	*n* = 8 (32%)	*n* = 2 (50%)
Concerns with blood clots	*n* = 1164 (26.1%)	*n* = 47 (20.1%)	*n* = 189 (21.0%)	*n* = 162 (21.7%)	*n* = 411 (28.5%)	*n* = 244 (33.2%)	*n* = 82 (29.1%)	*n* = 29 (31.2%)	*n* = 5 (20%)	*n* = 1 (25%)
Cultural/family concerns	*n* = 546 (12.2%)	*n* = 26 (11.1%)	*n* = 89 (9.9%)	*n* = 94 (12.6%)	*n* = 189 (13.1%)	*n* = 98 (13.3%)	*n* = 36 (12.8%)	*n* = 23 (24.7%)	*n* = 5 (20%)	*n* = 0 (0.0%)
Risk of fertility	*n* = 280 (6.3%)	*n* = 37 (15.8%)	*n* = 109 (12.1%)	*n* = 64 (8.6%)	*n* = 60 (4.2%)	*n* = 9 (1.2%)	*n* = 0 (0.0%)	*n* = 9 (9.7%)	*n* = 1 (4.0%)	*n* = 0 (0.0%)
Pregnancy/breastfeeding	*n* = 269 (6.0%)	*n* = 19 (8.1%)	*n* = 69 (7.7%)	*n* = 77 (10.3%)	*n* = 98 (6.8%)	*n* = 5 (0.7%)	*n* = 1 (0.4%)	*n* = 0 (0.0%)	*n* = 0 (0.0%)	*n* = 0 (0.0%)
Does not want it-no reason	*n* = 153 (3.4%)	*n* = 1 (0.4%)	*n* = 28 (3.1%)	*n* = 32 (4.3%)	*n* = 38 (2.6%)	*n* = 26 (3.5%)	*n* = 17 (6.0%)	*n* = 0 (0.0%)	*n* = 1 (4.0%)	*n* = 1 (25%)
Not comfortable or confident yet	*n* = 137 (3.1%)	*n* = 5 (2.1%)	*n* = 20 (2.2%)	*n* = 26 (3.5%)	*n* = 47 (3.3%)	*n* = 22 (3.0%)	*n* = 12 (4.3%)	*n* = 9 (9.7%)	*n* = 0 (0.0%)	*n* = 0 (0.0%)
Side effects	*n* = 87 (1.9%)	*n* = 2 (0.9%)	*n* = 26 (2.9%)	*n* = 22 (2.9%)	*n* = 19 (1.3%)	*n* = 14 (1.9%)	*n* = 4 (1.4%)	*n* = 5 (5.4%)	*n* = 0 (0.0%)	*n* = 0 (0.0%)
Not enough evidence	*n* = 78 (1.7%)	*n* = 3 (1.3%)	*n* = 18 (2.0%)	*n* = 10 (1.3%)	*n* = 25 (1.7%)	*n* = 17 (2.3%)	*n* = 4 (1.4%)	*n* = 0 (0.0%)	*n* = 0 (0.0%)	*n* = 0 (0.0%)
Health issues	*n* = 52 (1.2%)	*n* = 0 (0.0%)	*n* = 6 (0.7%)	*n* = 9 (1.2%)	*n* = 14 (1.0%)	*n* = 14 (1.9%)	*n* = 7 (2.5%)	*n* = 1 (1.1%)	*n* = 1 (4.0%)	*n* = 0 (0.0%)
Does not believe it is needed	*n* = 44 (1.0%)	*n* = 1 (0.4%)	*n* = 9 (1.0%)	*n* = 8 (1.1%)	*n* = 12 (0.8%)	*n* = 4 (0.5%)	*n* = 2 (0.7%)	*n* = 1 (1.1%)	*n* = 1 (4.0%)	*n* = 0 (0.0%)
Does not trust it	*n* = 33 (0.7%)	*n* = 3 (1.3%)	*n* = 5 (0.6%)	*n* = 10 (1.3%)	*n* = 8 (0.6%)	*n* = 3 (0.4%)	*n* = 3 (1.1%)	*n* = 7 (7.5%)	*n* = 0 (0.0%)	*n* = 0 (0.0%)
Anti-vaccine	*n* = 25 (0.6%)	*n* = 0 (0.0%)	*n* = 3 (0.3%)	*n* = 1 (0.1%)	*n* = 6 (0.4%)	*n* = 11 (1.5%)	*n* = 3 (1.1%)	*n* = 1 (1.1%)	*n* = 0 (0.0%)	*n* = 0 (0.0%)
Not had time	*n* = 24 (0.5%)	*n* = 1 (0.4%)	*n* = 8 (0.9%)	*n* = 5 (0.7%)	*n* = 7 (0.5%)	*n* = 2 (0.3%)	*n* = 1 (0.4%)	*n* = 1 (1.1%)	*n* = 0 (0.0%)	*n* = 0 (0.0%)
Scared	*n* = 24 (0.5%)	*n* = 0 (0.0%)	*n* = 2 (0.2%)	*n* = 5 (0.7%)	*n* = 7 (0.5%)	*n* = 3 (0.4%)	*n* = 1 (0.4%)	*n* = 0 (0.0%)	*n* = 2 (8%)	*n* = 0 (0.0%)
Needle phobia	*n* = 20 (0.4%)	*n* = 2 (0.9%)	*n* = 3 (0.3%)	*n* = 3 (0.4%)	*n* = 5 (0.3%)	*n* = 3 (0.4%)	*n* = 4 (1.4%)	*n* = 4 (4.3%)	*n* = 0 (0.0%)	*n* = 0 (0.0%)
No reason given	*n* = 18 (0.4%)	*n* = 0 (0.0%)	*n* = 10 (1.1%)	*n* = 3 (0.4%)	*n* = 2 (0.1%)	*n* = 1 (0.1%)	*n* = 2 (0.7%)	*n* = 0 (0.0%)	*n* = 0 (0.0%)	*n* = 0 (0.0%)
Allergies	*n* = 17 (0.4%)	*n* = 2 (0.9%)	*n* = 1 (0.1%)	*n* = 4 (0.5%)	*n* = 8 (0.6%)	*n* = 0 (0.0%)	*n* = 0 (0.0%)	*n* = 0 (0.0%)	*n* = 1 (4.0%)	*n* = 0 (0.0%)
Could not make an appointment	*n* = 17 (0.4%)	*n* = 1 (0.4%)	*n* = 2 (0.2%)	*n* = 2 (0.3%)	*n* = 6 (0.4%)	*n* = 4 (0.5%)	*n* = 1	*n* = 1 (1.1%)	*n* = 0 (0.0%)	*n* = 0 (0.0%)
Personal reasons	*n* = 14 (0.3%)	*n* = 1 (0.4%)	*n* = 4 (0.4%)	*n* = 2 (0.3%)	*n* = 6 (0.4%)	*n* = 1 (0.1%)	*n* = 0 (0.0%)	*n* = 1 (1.1%)	*n* = 0 (0.0%)	*n* = 0 (0.0%)
Adverse reaction previously	*n* = 12 (0.3%)	*n* = 0 (0.0%)	*n* = 1 (0.1%)	*n* = 3 (0.4%)	*n* = 3 (0.2%)	*n* = 4 (0.5%)	*n* = 0 (0.0%)	*n* = 0 (0.0%)	*n* = 0 (0.0%)	*n* = 0 (0.0%)
Had COVID	*n* = 8 (0.2%)	*n* = 0 (0.0%)	*n* = 0 (0.0%)	*n* = 3 (0.4%)	*n* = 2 (0.1%)	*n* = 2 (0.3%)	*n* = 1 (0.4%)	*n* = 1 (1.1%)	*n* = 0 (0.0%)	*n* = 0 (0.0%)
Does not want to be told what to do	*n* = 7 (0.2%)	*n* = 0 (0.0%)	*n* = 2 (0.2%)	*n* = 0 (0.0%)	*n* = 4 (0.3%)	*n* = 1 (0.1%)	*n* = 0 (0.0%)	*n* = 0 (0.0%)	*n* = 0 (0.0%)	*n* = 0 (0.0%)
Does not believe COVID exists	*n* = 5 (0.1%)	*n* = 0 (0.0%)	*n* = 1 (0.1%)	*n* = 0 (0.0%)	*n* = 3 (0.2%)	*n* = 1 (0.1%)	*n* = 0 (0.0%)	*n* = 0 (0.0%)	*n* = 0 (0.0%)	*n* = 0 (0.0%)
Wants specific brand of vaccine	*n* = 5 (0.1%)	*n* = 0 (0.0%)	*n* = 0 (0.0%)	*n* = 1 (0.1%)	*n* = 1 (0.1%)	*n* = 1 (0.1%)	*n* = 2 (0.7%)	*n* = 0 (0.0%)	*n* = 0 (0.0%)	*n* = 0 (0.0%)

**Table 4:** Reasons for hesitance by ethnicity, part 1.

Reason for Hesitation	Overall (*n* = 4464)	Arab % (*n* = 48)	Asian or Asian British-Chinese % (*n* = 116)	Asian or Asian British-Indian % (*n* = 259)	Asian or Asian British-Other Asian Background % (*n* = 94)	Asian or Asian British-Pakistani % (*n* = 218)	Black or Black British-African % (*n* = 908)	Black or Black British-Caribbean % (*n* = 480)	Black or Black British-Other Black Background % (*n* = 57)	Mixed-Other Mixed Background % (*n* = 37)
Lack of understanding	*n* = 1425 (31.9%)	*n* = 15 (31.3%)	*n* = 48 (41.4%)	*n* = 73 (28.2%)	*n* = 23 (24.5%)	*n* = 38 (17.4%)	*n* = 288 (31.7%)	*n* = 126 (26.3%)	*n* = 11 (19.3%)	*n* = 10 (27.0%)
Concerns with blood clots	*n* = 1164 (26.1%)	*n* = 5 (10.4%)	*n* = 41 (35.3%)	*n* = 84 (32.4%)	*n* = 38 (40.4%)	*n* = 57 (26.1%)	*n* = 241 (26.5%)	*n* = 100 (20.8%)	*n* = 26 (45.6%)	*n* = 15 (40.5%)
Cultural/family concerns	*n* = 546 (12.2%)	*n* = 18 (37.5%)	*n* = 15 (12.9%)	*n* = 46 (17.8%)	*n* = 16 (17.0%)	*n* = 80 (36.7%)	*n* = 142 (15.6%)	*n* = 67 (14.0%)	*n* = 5 (8.8%)	*n* = 4 (10.8%)
Risk of fertility	*n* = 280 (6.3%)	*n* = 1 (2.1%)	*n* = 4 (3.4%)	*n* = 14 (5.4%)	*n* = 4 (4.3%)	*n* = 20 (9.2%)	*n* = 62 (6.8%)	*n* = 16 (3.3%)	*n* = 3 (5.3%)	*n* = 0 (0.0%)
Pregnancy/breastfeeding	*n* = 269 (6.0%)	*n* = 3 (6.3%)	*n* = 4 (3.4%)	*n* = 11 (4.2%)	*n* = 2 (2.1%)	*n* = 15 (6.9%)	*n* = 31 (3.4%)	*n* = 13 (2.7%)	*n* = 4 (7.0%)	*n* = 4 (10.8%)
Does not want it-no reason	*n* = 153 (3.4%)	*n* = 3 (6.3%)	*n* = 0 (0.0%)	*n* = 8 (3.1%)	*n* = 3 (3.2%)	*n* = 2 (0.9%)	*n* = 24 (2.6%)	*n* = 43 (9.0%)	*n* = 1 (1.8%)	*n* = 1 (2.7%)
Not comfortable or confident yet	*n* = 137 (3.1%)	*n* = 0 (0.0%)	*n* = 0 (0.0%)	*n* = 3 (1.2%)	*n* = 0 (0.0%)	*n* = 0 (0.0%)	*n* = 23 (2.5%)	*n* = 37 (7.7%)	*n* = 4 (7.0%)	*n* = 2 (5.4%)
Side effects	*n* = 87 (1.9%)	*n* = 0 (0.0%)	*n* = 0 (0.0%)	*n* = 6 (2.3%)	*n* = 0 (0.0%)	*n* = 1 (0.5%)	*n* = 23 (2.5%)	*n* = 5 (1.0%)	*n* = 0 (0.0%)	*n* = 0 (0.0%)
Not enough evidence	*n* = 78 (1.7%)	*n* = 2 (4.2%)	*n* = 0 (0.0%)	*n* = 0 (0.0%)	*n* = 1 (1.1%)	*n* = 1 (0.5%)	*n* = 9 (1.0%)	*n* = 16 (3.3%)	*n* = 1 (1.8%)	*n* = 1 (2.7%)
Health issues	*n* = 52 (1.2%)	*n* = 0 (0.0%)	*n* = 0 (0.0%)	*n* = 3 (1.2%)	*n* = 2 (2.1%)	*n* = 2 (0.9%)	*n* = 11 (1.2%)	*n* = 10 (2.1%)	*n* = 1 (1.8%)	*n* = 0 (0.0%)
Does not believe it is needed	*n* = 44 (1.0%)	*n* = 0 (0.0%)	*n* = 0 (0.0%)	*n* = 3 (1.2%)	*n* = 2 (2.1%)	*n* = 0 (0.0%)	*n* = 11 (1.2%)	*n* = 3 (0.6%)	*n* = 1 (4.0%)	*n* = 0 (0.0%)
Does not trust it	*n* = 33 (0.7%)	*n* = 1 (2.1%)	*n* = 1 (0.9%)	*n* = 0 (0.0%)	*n* = 0 (0.0%)	*n* = 0 (0.0%)	*n* = 4 (0.4%)	*n* = 7 (1.7%)	*n* = 0 (0.0%)	*n* = 0 (0.0%)
Anti-vaccine	*n* = 25 (0.6%)	*n* = 0 (0.0%)	*n* = 0 (0.0%)	*n* = 0 (0.0%)	*n* = 0 (0.0%)	*n* = 0 (0.0%)	*n* = 7 (0.8%)	*n* = 3 (0.6%)	*n* = 0 (0.0%)	*n* = 0 (0.0%)
Not had time	*n* = 24 (0.5%)	*n* = 0 (0.0%)	*n* = 1 (0.9%)	*n* = 0 (0.0%)	*n* = 0 (0.0%)	*n* = 0 (0.0%)	*n* = 7 (0.8%)	*n* = 8 (1.7%)	*n* = 0 (0.0%)	*n* = 0 (0.0%)
Scared	*n* = 24 (0.5%)	*n* = 0 (0.0%)	*n* = 0 (0.0%)	*n* = 0 (0.0%)	*n* = 1 (1.1%)	*n* = 0 (0.0%)	*n* = 5 (0.6%)	*n* = 5 (1.0%)	*n* = 0 (0.0%)	*n* = 0 (0.0%)
Needle phobia	*n* = 20 (0.4%)	*n* = 0 (0.0%)	*n* = 0 (0.0%)	*n* = 1 (0.4%)	*n* = 0 (0.0%)	*n* = 1 (0.5%)	*n* = 1 (0.1%)	*n* = 5 (1.0%)	*n* = 0 (0.0%)	*n* = 0 (0.0%)
No reason given	*n* = 18 (0.4%)	*n* = 0 (0.0%)	*n* = 1 (0.9%)	*n* = 1 (0.4%)	*n* = 1 (1.1%)	*n* = 0 (0.0%)	*n* = 5 (0.6%)	*n* = 2 (0.4%)	*n* = 0 (0.0%)	*n* = 0 (0.0%)
Allergies	*n* = 17 (0.4%)	*n* = 0 (0.0%)	*n* = 0 (0.0%)	*n* = 2 (0.8%)	*n* = 0 (0.0%)	*n* = 1 (0.5%)	*n* = 2 (0.2%)	*n* = 3 (0.6%)	*n* = 0 (0.0%)	*n* = 0 (0.0%)
Could not make an appointment	*n* = 17 (0.4%)	*n* = 0 (0.0%)	*n* = 0 (0.0%)	*n* = 0 (0.0%)	*n* = 1 (1.1%)	*n* = 0 (0.0%)	*n* = 1 (0.1%)	*n* = 1 (0.2%)	*n* = 0 (0.0%)	*n* = 0 (0.0%)
Personal reasons	*n* = 14 (0.3%)	*n* = 0 (0.0%)	*n* = 0 (0.0%)	*n* = 1 (0.4%)	*n* = 0 (0.0%)	*n* = 0 (0.0%)	*n* = 4 (0.4%)	*n* = 3 (0.6%)	*n* = 0 (0.0%)	*n* = 0 (0.0%)
Adverse reaction previously	*n* = 12 (0.3%)	*n* = 0 (0.0%)	*n* = 0 (0.0%)	*n* = 3 (0.4%)	*n* = 0 (0.0%)	*n* = 0 (0.0%)	*n* = 2 (0.2%)	*n* = 4 (0.8%)	*n* = 0 (0.0%)	*n* = 0 (0.0%)
Had COVID	*n* = 8 (0.2%)	*n* = 0 (0.0%)	*n* = 0 (0.0%)	*n* = 0 (0.0%)	*n* = 0 (0.0%)	*n* = 0 (0.0%)	*n* = 0 (0.0%)	*n* = 1 (0.2%)	*n* = 0 (0.0%)	*n* = 0 (0.0%)
Does not want to be told what to do	*n* = 7 (0.2%)	*n* = 0 (0.0%)	*n* = 0 (0.0%)	*n* = 1 (0.4%)	*n* = 0 (0.0%)	*n* = 0 (0.0%)	*n* = 2 (0.2%)	*n* = 1 (0.2%)	*n* = 0 (0.0%)	*n* = 0 (0.0%)
Does not believe COVID exists	*n* = 5 (0.1%)	*n* = 0 (0.0%)	*n* = 0 (0.0%)	*n* = 0 (0.0%)	*n* = 0 (0.0%)	*n* = 0 (0.0%)	*n* = 2 (0.2%)	*n* = 0 (0.0%)	*n* = 0 (0.0%)	*n* = 0 (0.0%)
Wants specific brand of vaccine	*n* = 5 (0.1%)	*n* = 0 (0.0%)	*n* = 0 (0.0%)	*n* = 0 (0.0%)	*n* = 0 (0.0%)	*n* = 0 (0.0%)	*n* = 1 (0.1%)	*n* = 1 (0.2%)	*n* = 0 (0.0%)	*n* = 0 (0.0%)

**Table 5:** Reasons for hesitance by ethnicity, part 2.

Reason for Hesitation	Overall (*n* = 4464)	Mixed-White & Asian % (*n* = 57)	Mixed-White & Black African % (*n* = 123)	Mixed-White & Black Caribbean % (*n* = 109)	Other Ethnic Group % (*n* = 60)	Prefer not to Say % (*n* = 245)	White-British % (*n* = 1290)	White-Gypsy or Irish Traveller % (*n* = 17)	White-Irish % (*n* = 81)	White-Other % (*n* = 265)
Lack of understanding	*n* = 1425 (31.9%)	*n* = 13 (22.8%)	*n* = 31 (25.2%)	*n* = 32 (29.4%)	*n* = 25 (41.7%)	*n* = 95 (38.8%)	*n* = 464 (36.0%)	*n* = 5 (29.4%)	*n* = 28 (34.6%)	*n* = 100 (37.7%)
Concerns with blood clots	*n* = 1164 (26.1%)	*n* = 21 (36.8%)	*n* = 43 (35.0%)	*n* = 31 (28.4%)	*n* = 11 (18.3%)	*n* = 60 (24.5%)	*n* = 33 (25.0%)	*n* = 4 (23.5%)	*n* = 18 (22.2%)	*n* = 47 (17.7%)
Cultural/family concerns	*n* = 546 (12.2%)	*n* = 8 (14.0%)	*n* = 15 (12.2%)	*n* = 12 (11.0%)	*n* = 9 (15.0%)	*n* = 25 (10.2%)	*n* = 57 (4.4%)	*n* = 3 (17.6%)	*n* = 5 (6.2%)	*n* = 19 (7.2%)
Risk of fertility	*n* = 280 (6.3%)	*n* = 5 (8.8%)	*n* = 10 (8.1%)	*n* = 16 (14.7%)	*n* = 1 (1.7%)	*n* = 13 (5.3%)	*n* = 86 (6.7%)	*n* = 1 (5.9%)	*n* = 10 (12.3%)	*n* = 14 (5.3%)
Pregnancy/breastfeeding	*n* = 269 (6.0%)	*n* = 5 (8.8%)	*n* = 8 (6.5%)	*n* = 2 (1.8%)	*n* = 5 (8.3%)	*n* = 16 (6.5%)	*n* = 110 (8.5%)	*n* = 4 (23.5%)	*n* = 8 (9.9%)	*n* = 24 (9.1%)
Does not want it-no reason	*n* = 153 (3.4%)	*n* = 0 (0.0%)	*n* = 2 (1.6%)	*n* = 3 (2.8%)	*n* = 0 (0.0%)	*n* = 6 (2.4%)	*n* = 46 (3.6%)	*n* = 0 (0.0%)	*n* = 3 (3.7%)	*n* = 8 (3.0%)
Not comfortable or confident yet	*n* = 137 (3.1%)	*n* = 1 (1.8%)	*n* = 4 (3.3%)	*n* = 2 (1.8%)	*n* = 3 (5.0%)	*n* = 3 (1.2%)	*n* = 39 (3.0%)	*n* = 0 (0.0%)	*n* = 3 (3.7%)	*n* = 13 (4.9%)
Side effects	*n* = 87 (1.9%)	*n* = 0 (0.0%)	*n* = 2 (1.6%)	*n* = 0 (0.0%)	*n* = 0 (0.0%)	*n* = 1 (0.4%)	*n* = 41 (3.2%)	*n* = 0 (0.0%)	*n* = 1 (1.2%)	*n* = 7 (2.6%)
Not enough evidence	*n* = 78 (1.7%)	*n* = 2 (3.5%)	*n* = 3 (2.4%)	*n* = 3 (2.8%)	*n* = 2 (3.3%)	*n* = 2 (0.8%)	*n* = 22 (1.7%)	*n* = 0 (0.0%)	*n* = 0 (0.0%)	*n* = 13 (4.9%)
Health issues	*n* = 52 (1.2%)	*n* = 0 (0.0%)	*n* = 1 (0.8%)	*n* = 2 (1.8%)	*n* = 0 (0.0%)	*n* = 4 (1.6%)	*n* = 12 (0.9%)	*n* = 0 (0.0%)	*n* = 0 (0.0%)	*n* = 4 (1.5%)
Does not believe it is needed	*n* = 44 (1.0%)	*n* = 0 (0.0%)	*n* = 2 (1.6%)	*n* = 1 (0.9%)	*n* = 0 (0.0%)	*n* = 2 (0.8%)	*n* = 15 (1.2%)	*n* = 0 (0.0%)	*n* = 1 (1.2%)	*n* = 3 (1.1%)
Does not trust it	*n* = 33 (0.7%)	*n* = 3 (1.3%)	*n* = 1 (0.8%)	*n* = 1 (0.9%)	*n* = 0 (0.0%)	*n* = 6 (2.4%)	*n* = 8 (0.6%)	*n* = 0 (0.0%)	*n* = 1 (1.2%)	*n* = 2 (0.8%)
Anti-vaccine	*n* = 25 (0.6%)	*n* = 0 (0.0%)	*n* = 1 (0.8%)	*n* = 0 (0.0%)	*n* = 0 (0.0%)	*n* = 3 (1.2%)	*n* = 10 (0.6%)	*n* = 0 (0.0%)	*n* = 0 (0.0%)	*n* = 1 (0.4%)
Not had time	*n* = 24 (0.5%)	*n* = 0 (0.0%)	*n* = 0 (0.0%)	*n* = 0 (0.0%)	*n* = 0 (0.0%)	*n* = 2 (0.8%)	*n* = 5 (0.4%)	*n* = 0 (0.0%)	*n* = 1 (1.2%)	*n* = 0 (0.0%)
Scared	*n* = 24 (0.5%)	*n* = 1 (1.8%)	*n* = 0 (0.0%)	*n* = 0 (0.0%)	*n* = 0 (0.0%)	*n* = 1 (0.4%)	*n* = 10 (0.8%)	*n* = 0 (0.0%)	*n* = 0 (0.0%)	*n* = 1 (0.4%)
Needle phobia	*n* = 20 (0.4%)	*n* = 0 (0.0%)	*n* = 0 (0.0%)	*n* = 0 (0.0%)	*n* = 0 (0.0%)	*n* = 0 (0.0%)	*n* = 11 (0.9%)	*n* = 0 (0.0%)	*n* = 1 (1.2%)	*n* = 0 (0.0%)
No reason given	*n* = 18 (0.4%)	*n* = 0 (0.0%)	*n* = 0 (0.0%)	*n* = 1 (0.9%)	*n* = 1 (1.7%)	*n* = 1 (0.4%)	*n* = 5 (0.4%)	*n* = 0 (0.0%)	*n* = 0 (0.0%)	*n* = 0 (0.0%)
Allergies	*n* = 17 (0.4%)	*n* = 0 (0.0%)	*n* = 0 (0.0%)	*n* = 0 (0.0%)	*n* = 0 (0.0%)	*n* = 2 (0.8%)	*n* = 5 (0.4%)	*n* = 0 (0.0%)	*n* = 0 (0.0%)	*n* = 1 (0.4%)
Could not make an appointment	*n* = 17 (0.4%)	*n* = 0 (0.0%)	*n* = 0 (0.0%)	*n* = 0 (0.0%)	*n* = 2 (3.3%)	*n* = 1 (0.4%)	*n* = 6 (0.5%)	*n* = 0 (0.0%)	*n* = 1 (1.2%)	*n* = 4 (1.5%)
Personal reasons	*n* = 14 (0.3%)	*n* = 0 (0.0%)	*n* = 0 (0.0%)	*n* = 1 (0.9%)	*n* = 0 (0.0%)	*n* = 0 (0.0%)	*n* = 4 (0.3%)	*n* = 0 (0.0%)	*n* = 0 (0.0%)	*n* = 0 (0.0%)
Adverse reaction previously	*n* = 12 (0.3%)	*n* = 0 (0.0%)	*n* = 0 (0.0%)	*n* = 0 (0.0%)	*n* = 0 (0.0%)	*n* = 0 (0.0%)	*n* = 5 (0.4%)	*n* = 0 (0.0%)	*n* = 0 (0.0%)	*n* = 0 (0.0%)
Had COVID	*n* = 8 (0.2%)	*n* = 0 (0.0%)	*n* = 0 (0.0%)	*n* = 1 (0.9%)	*n* = 0 (0.0%)	*n* = 0 (0.0%)	*n* = 3 (0.2%)	*n* = 0 (0.0%)	*n* = 0 (0.0%)	*n* = 3 (1.1%)
Does not want to be told what to do	*n* = 7 (0.2%)	*n* = 0 (0.0%)	*n* = 0 (0.0%)	*n* = 0 (0.0%)	*n* = 0 (0.0%)	*n* = 1 (0.4%)	*n* = 2 (0.2%)	*n* = 0 (0.0%)	*n* = 0 (0.0%)	*n* = 0 (0.0%)
Does not believe COVID exists	*n* = 5 (0.1%)	*n* = 0 (0.0%)	*n* = 0 (0.0%)	*n* = 0 (0.0%)	*n* = 0 (0.0%)	*n* = 1 (0.4%)	*n* = 2 (0.2%)	*n* = 0 (0.0%)	*n* = 0 (0.0%)	*n* = 0 (0.0%)
Wants specific brand of vaccine	*n* = 5 (0.1%)	*n* = 0 (0.0%)	*n* = 0 (0.0%)	*n* = 1 (0.9%)	*n* = 1 (1.7%)	*n* = 0 (0.0%)	*n* = 0 (0.0%)	*n* = 0 (0.0%)	*n* = 0 (0.0%)	*n* = 1 (0.4%)

**Table 6:** Initial hesitance and final agreement to vaccination by demographic.

*n* = 8539	Initial Hesitation (*n* = 4464)	Agreed to Vaccination (*n* = 6093)	Conversion from Initial Hesitation to Agreement to Vaccination
Overall (*n* = 8539)	*n* = 4464 (52.3%)	*n* = 6094 (71.4%)	*n* = 1630 (19.1%)
Bexley (*n* = 2030)	*n* = 1190 (58.6%)	*n* = 1636 (80.6%)	*n* = 446 (22.0%)
Bromley (*n* = 709)	*n* = 284 (40.1%)	*n* = 594 (83.8%)	*n* = 310 (43.7%)
Greenwich (*n* = 2126)	*n* = 1067 (50.2%)	*n* = 1593 (74.9%)	*n* = 526 (24.7%)
Lambeth (*n* = 798)	*n* = 584 (73.2%)	*n* = 151 (18.9%)	*n* = −433 (−54.3%)
Lewisham (*n* = 2173)	*n* = 1109 (51.0%)	*n* = 1507 (69.4%)	*n* = 399 (18.4%)
Southwark (*n* = 703)	*n* = 230 (32.7%)	*n* = 612 (87.1%)	*n* = 382 (54.4%)
Female (*n* = 4655)	*n* = 2466 (53%)	*n* = 3322 (71.4%)	*n* = 856 (18.4%)
Male (*n* = 3862)	*n* = 1992 (51.6%)	*n* = 2758 (71.6%)	*n* = 766 (19.8%)
Transgender (*n* = 22)	*n* = 6 (27.3%)	*n* = 14 (63.6%)	*n* = 8 (36.3%)
18–20 (*n* = 591)	*n* = 234 (39.6%)	*n* = 497 (84.1%)	*n* = 263 (44.5%)
21–30 (*n* = 1522)	*n* = 901 (59.2)	*n* = 1059 (69.6%)	*n* = 158 (10.4%)
31–40 (*n* = 1308)	*n* = 747 (57.1%)	*n* = 823 (62.9%)	*n* = 76 (5.8%)
41–50 (*n* = 2503)	*n* = 1442 (57.6%)	*n* = 1747 (69.8%)	*n* = 305 (12.2%)
51–60 (*n* = 1418)	*n* = 736 (51.9%)	*n* = 1053 (74.3%)	*n* = 317 (22.4%)
61–70 (*n* = 709)	*n* = 282 (39.8%)	*n* = 522 (73.6%)	*n* = 240 (33.8%)
71–80 (*n* = 346)	*n* = 93 (26.9%)	*n* = 275 (79.5%)	*n* = 182 (52.6%)
81–90 (*n* = 125)	*n* = 25 (20.0%)	*n* = 105 (84%)	*n* = 80 (64.0%)
91–100 (*n* = 17)	*n* = 4 (23.5%)	*n* = 13 (76.5%)	*n* = 9 (53.0%)
Arab (*n* = 88)	*n* = 48 (54.5%)	*n* = 65 (73.9%)	*n* = 17 (19.4%)
Asian or Asian British-Chinese (*n* = 245)	*n* = 116 (47.3%)	*n* = 190 (77.6%)	*n* = 74 (30.3%)
Asian or Asian British-Indian (*n* = 500)	*n* = 259 (51.8%)	*n* = 410 (82%)	*n* = 151 (30.2%)
Asian or Asian British-Other Asian background (*n* = 138)	*n* = 94 (68.1%)	*n* = 118 (85.5%)	*n* = 24 (17.4%)
Asian or Asian British-Pakistani (*n* = 333)	*n* = 218 (65.5%)	*n* = 265 (79.6%)	*n* = 47 (14.1%)
Black or Black British-African (*n* = 1447)	*n* = 908 (62.8%)	*n* = 992 (68.6%)	*n* = 84 (5.8%)
Black or Black British-Caribbean (*n* = 692)	*n* = 480 (69.4%)	*n* = 348 (50.3%)	*n* = −132 (−19.1%)
Black or Black British-Other Black background (*n* = 95)	*n* = 57 (60.0%)	*n* = 74 (77.9%)	*n* = 17 (17.9%)
Mixed-Other mixed background (*n* = 65)	*n* = 37 (56.9%)	*n* = 43 (66.2%)	*n* = 6 (9.3%)
Mixed-White & Asian (*n* = 157)	*n* = 57 (36.3%)	*n* = 127 (80.9%)	*n* = 70 (44.6%)
Mixed-White &Black African (*n* = 226)	*n* = 123 (54.4%)	*n* = 148 (65.5%)	*n* = 25 (11.1%)
Mixed-White &Black Caribbean (*n* = 235)	*n* = 109 (46.4%)	*n* = 161 (68.5%)	*n* = 52 (22.1%)
Other ethnic group (*n* = 119)	*n* = 60 (50.4%)	*n* = 92 (77.3%)	*n* = 32 (26.9%)
Prefer not to say (*n* = 499)	*n* = 245 (49.1%)	*n* = 346 (69.3%)	*n* = 101 (20.2%)
White-British (*n* = 2854)	*n* = 1290 (45.2%)	*n* = 2053 (71.9%)	*n* = 763 (26.7%)
White-Gypsy or Irish traveller (*n* = 87)	*n* = 17 (19.5%)	*n* = 83 (95.4%)	*n* = 66 (75.9%)
White-Irish (*n* = 228)	*n* = 81 (35.5%)	*n* = 190 (83.3%)	*n* = 109 (47.8%)
White-other (*n* = 531)	*n* = 265 (49.9%)	*n* = 389 (73.3%)	*n* = 124 (23.4%)

**Table 7:** Overall outcomes.

Outcome	Those Who Agreed to Vaccination (*n* = 6094)	Those Who Did Not Agreed to Vaccination (*n* = 2445)
Signposted to LVS	*n* = 2090 (34.3%)	*n* = 32 (1.3%)
Vaccinated at the pharmacy	*n* = 1223 (20.1%)	*n* = 0 (0%)
Signposted to NBS	*n* = 1171(19.2%)	*n* = 21 (0.9%)
Signposted to pharmacy vaccination site	*n* = 430 (7.1%)	*n* = 0 (0%)
Returning to the pharmacy for the vaccine at a later date	*n* = 259 (4.3%)	*n* = 158 (6.5%)
Booked at LVS	*n* = 255 (4.2%)	*n* = 5 (0.2%)
Booked on NBS	*n* = 243 (4%)	*n* = 2 (0.1%)
Already had both doses	*n* = 155 (2.5%)	*n* = 150 (6.1%)
No outcome given	*n* = 72 (1.2%)	*n* = 74 (3.0%)
Will book later	*n* = 69 (1.1%)	*n* = 13 (0.5%)
2nd dose booked	*n* = 50 (0.8%)	*n* = 2 (0.1%)
Did not agree to vaccination	*n* = 15 (0.2%)	*n* = 759 (31.0%)
Wanted specific brand of vaccine	*n* = 12 (0.2%)	*n* = 8 (0.3%)
More information given	*n* = 12 (0.2%)	*n* = 712 (29.1%)
Currently pregnant/breastfeeding. Will consider later	*n* = 8 (0.1%)	*n* = 18 (0.7%)
2nd dose booked on NBS	*n* = 8 (0.1%)	*n* = 0 (0%)
Signposted to GP	*n* = 7 (0.1%)	*n* = 64 (2.6%)
2nd dose booked at LVS	*n* = 5 (0.1%)	*n* = 0 (0%)
Will think about it	*n* = 6 (0.1%)	*n* = 131 (5.4%)
Concerns over safety	*n* = 2 (0%)	*n* = 202 (8.3%)
Medically exempt	*n* = 1 (0%)	*n* = 10 (0.4%)
Needle phobia	*n* = 1 (0%)	*n* = 11 (0.4%)
Community outreach required	*n* = 0 (0%)	*n* = 19 (0.8%)
Had COVID so does not believe vaccine is necessary	*n* = 0 (0%)	*n* = 6 (0.2%)
Language specific information required	*n* = 0 (0%)	*n* = 17 (0.7%)
Review vaccine status at next visit	*n* = 0 (0%)	*n* = 24 (1.0%)
Refused 2nd dose due to reaction to 1st	*n* = 0 (0%)	*n* = 7 (0.3%)

**Table 8:** Top 7 outcomes for those who agreed to vaccination by demographic.

	SignPosted to LVS (*n* = 2122)	Vaccinated at the Pharmacy (*n* = 1223)	SignPosted to NBS (*n* = 1192)	Did Not Agree to Vaccination (*n* = 774)	More Information Given (*n* = 724)	SignPosted to a Pharmacy Vaccination Site (*n* = 430)	Returning to the Pharmacy for the Vaccine at a Later Date (*n* = 417)
Overall (*n* = 8539)	*n* = 2122 (24.9%)	*n* = 1223 (14.3%)	*n* = 1192 (14.0%)	*n* = 774 (9.1%)	*n* = 724 (8.5%)	*n* = 430 (5.0%)	*n* = 417 (4.9%)
Female (*n* = 4655)	*n* = 1163 (25.0%)	*n* = 588 (12.6%)	*n* = 661 (14.2%)	*n* = 402 (8.6%)	*n* = 389 (8.4%)	*n* = 286 (6.1%)	*n* = 254 (5.5%)
Male (*n* = 3862)	*n* = 957 (24.8%)	*n* = 633 (16.4%)	*n* = 530 (13.7%)	*n* = 371 (9.6%)	*n* = 330 (8.5%)	*n* = 140 (3.6%)	*n* = 160 (4.1%)
Transgender (*n* = 22)	*n* = 2 (9.1%)	*n* = 2 (9.1%)	*n* = 1 (4.5%)	*n* = 1 (4.5%)	*n* = 4 (18.2%)	*n* = 4 (18.2%)	*n* = 3 (13.6%)
18–20 (*n* = 591)	*n* = 136 (23.0%)	*n* = 132 (22.3%)	*n* = 97 (16.4%)	*n* = 31 (5.2%)	*n* = 24 (4.1%)	*n* = 26 (4.4%)	*n* = 19 (3.2%)
21–30 (*n* = 1522)	*n* = 268 (17.6%)	*n* = 253 (16.6%)	*n* = 315 (20.7%)	*n* = 176 (11.6%)	*n* = 164 (10.8%)	*n* = 50 (3.3%)	*n* = 63 (4.1%)
31–40 (*n* = 1308)	*n* = 222 (17.0%)	*n* = 223 (17.0%)	*n* = 134 (10.2%)	*n* = 169 (12.9%)	*n* = 159 (12.1%)	*n* = 64 (4.9%)	*n* = 69 (5.3%)
41–50 (*n* = 2503)	*n* = 580 (23.2%)	*n* = 380 (15.2%)	*n* = 358 (14.3%)	*n* = 207 (8.3%)	*n* = 222 (8.9%)	*n* = 104 (4.2%)	*n* = 163 (6.5%)
51–60 (*n* = 1418)	*n* = 425 (30.0%)	*n* = 169 (11.9%)	*n* = 193 (13.6%)	*n* = 115 (8.1%)	*n* = 73 (5.1%)	*n* = 99 (7.0%)	*n* = 74 (5.2%)
61–70 (*n* = 709)	*n* = 268 (37.8%)	*n* = 47 (6.6%)	*n* = 53 (7.5%)	*n* = 50 (7.1%)	*n* = 56 (7.9%)	*n* = 58 (8.2%)	*n* = 26 (3.7%)
71–80 (*n* = 346)	*n* = 159 (46.0%)	*n* = 18 (5.2%)	*n* = 24 (6.9%)	*n* = 19 (5.5%)	*n* = 21 (6.1%)	*n* = 23 (6.6%)	*n* = 2 (0.5%)
81–90 (*n* = 125)	*n* = 57 (46.0%)	*n* = 1 (0.8%)	*n* = 15 (12.1%)	*n* = 4 (3.2%)	*n* = 5 (4.0%)	*n* = 4 (3.2%)	*n* = 1 (0.8%)
91–100 (*n* = 17)	*n* = 7 (41.2%)	*n* = 0 (0%)	*n* = 3 (17.6%)	*n* = 3 (17.6%)	*n* = 0 (0%)	*n* = 2 (11.8%)	*n* = 0 (0%)
Arab (*n* = 88)	*n* = 16 (18.2%)	*n* = 14 (15.9%)	*n* = 11 (12.5%)	*n* = 11 (12.5%)	*n* = 3 (3.4%)	*n* = 11 (12.5%)	*n* = 5 (5.7%)
Asian or Asian British-Chinese (*n* = 245)	*n* = 32 (13.1%)	*n* = 45 (18.4%)	*n* = 57 (23.3%)	*n* = 16 (6.5%)	*n* = 17 (6.9%)	*n* = 12 (4.9%)	*n* = 11 (4.5%)
Asian or Asian British-Indian (*n* = 500)	*n* = 139 (27.8%)	*n* = 71 (14.2%)	*n* = 95 (19%)	*n* = 17 (3.4%)	*n* = 40 (8.0%)	*n* = 23 (4.6%)	*n* = 23 (4.6%)
Asian or Asian British-Other Asian background (*n* = 138)	*n* = 19 (13.8%)	*n* = 31 (22.5%)	*n* = 63 (45.7%)	*n* = 13 (9.4%)	*n* = 2 (1.4%)	*n* = 1 (0.7%)	*n* = 3 (2.2%)
Asian or Asian British-Pakistani (*n* = 333)	*n* = 56 (16.8%)	*n* = 22 (6.6%)	*n* = 112 (33.6%)	*n* = 26 (7.8%)	*n* = 19 (5.7%)	*n* = 19 (5.7%)	*n* = 17 (5.1%)
Black or Black British-African (*n* = 1447)	*n* = 346 (23.9%)	*n* = 231 (16.0%)	*n* = 230 (15.9%)	*n* = 123 (8.5%)	*n* = 169 (11.7%)	*n* = 17 (1.2%)	*n* = 61 (4.2%)
Black or Black British-Caribbean (*n* = 692)	*n* = 174 (25.1%)	*n* = 32 (4.6%)	*n* = 58 (8.4%)	*n* = 101 (14.6%)	*n* = 99 (14.3%)	*n* = 14 (2.0%)	*n* = 13 (1.9%)
Black or Black British-Other Black background (*n* = 95)	*n* = 12 (12.6%)	*n* = 25 (26.3%)	*n* = 24 (25.3%)	*n* = 10 (10.5%)	*n* = 3 (3.2%)	*n* = 0 (0.0%)	*n* = 2 (2.1%)
Mixed-Other mixed background (*n* = 65)	*n* = 7 (10.8%)	*n* = 14 (21.5%)	*n* = 13 (20.0%)	*n* = 9 (13.8%)	*n* = 2 (3.1%)	*n* = 1 (1.5%)	*n* = 3 (4.6%)
Mixed-White & Asian (*n* = 157)	*n* = 17 (10.8%)	*n* = 12 (7.6%)	*n* = 24 (15.3%)	*n* = 8 (5.1%)	*n* = 5 (3.2%)	*n* = 29 (18.5%)	*n* = 19 (12.1%)
Mixed-White &Black African (*n* = 226)	*n* = 26 (11.5%)	*n* = 19 (8.4%)	*n* = 20 (8.8%)	*n* = 29 (12.8%)	*n* = 19 (8.4%)	*n* = 44 (19.5%)	*n* = 29 (12.8%)
Mixed-White &Black Caribbean (*n* = 235)	*n* = 32 (13.6%)	*n* = 13 (5.5%)	*n* = 17 (7.2%)	*n* = 33 (14.0%)	*n* = 23 (9.8%)	*n* = 59 (25.1%)	*n* = 15 (6.4%)
Other ethnic group (*n* = 119)	*n* = 21 (17.6%)	*n* = 45 (37.8%)	*n* = 10 (8.4%)	*n* = 11 (9.2%)	*n* = 4 (3.4%)	*n* = 0 (0.0%)	*n* = 8 (6.7%)
Prefer not to say (*n* = 499)	*n* = 107 (21.4%)	*n* = 94 (18.8%)	*n* = 74 (14.8%)	*n* = 31 (6.2%)	*n* = 59 (11.8%)	*n* = 20 (4.0%)	*n* = 26 (5.2%)
White-British (*n* = 2854)	*n* = 958 (33.6%)	*n* = 360 (12.6%)	*n* = 327 (11.5%)	*n* = 265 (9.3%)	*n* = 236 (8.3%)	*n* = 82 (2.9%)	*n* = 136 (4.8%)
White-Gypsy or Irish traveller (*n* = 87)	*n* = 16 (18.4%)	*n* = 0 (0.0%)	*n* = 3 (3.4%)	*n* = 1 (1.1%)	*n* = 1 (1.1%)	*n* = 56 (64.4%)	*n* = 1 (1.1%)
White-Irish (*n* = 228)	*n* = 63 (27.6%)	*n* = 23 (10.1%)	*n* = 25 (11.1%)	*n* = 9 (3.9%)	*n* = 9 (3.9%)	*n* = 40 (17.5%)	*n* = 14 (6.1%)
White-other (*n* = 531)	*n* = 81 (15.3%)	*n* = 172 (32.4%)	*n* = 29 (5.5%)	*n* = 61 (11.5%)	*n* = 14 (2.6%)	*n* = 2 (0.4%)	*n* = 31 (5.8%)

**Table 9:** Pharmacist perceptions of the service. Mean score out of 10.

Question	Mean Score Out of 10
Required knowledge of where to signpost patients	8.5
Confidence to run the service	8.3
Knowledge to run the service	8.1
Patients understand the role of community pharmacy in public health promotion	7.9
The service supports patient understanding	7.7
PharmOutcomes in easy to use	7.3
Training received	7.2
Support available from commissioners	6.7
Resources needed	6.5
Patients are happy to discuss vaccination	6.1
Promotion material received	5.5

## Data Availability

The data presented in this study are available on request from the corresponding author. The data are not publicly available due to patient confidentiality issues.

## Appendix A. Interview Questions for Pharmacies

1. Please can you tell me about the motivation for the pharmacy to participate in the COVID champion service?
2. Can you tell me about how many of the team were engaged in the COVID champion service and how?
3. Overall, how does the pharmacy team feel about the service?
4. Please describe the process used in your pharmacy to identify potential participants
5. Overall, how have patients perceived the service?
6. Please can you share the biggest areas of feedback you had from patients that made them hesitant to have the COVID vaccination, and how did you overcome these?
7. Are you able to share a case study of one patient and their journey using the service?
8. What impact do you believe the service made on the local community?
9. How well do you believe the service supported patient understanding of the benefits of receiving a COVID vaccination?
10. If the service was going to be continued, what further support would you want or need to ensure the continuation of the service? What changes would you make if the service was to be rolled out?
11. Coming to the end of the service, looking back what were the barriers to delivery and what were the enablers?
12. What other services do you think community pharmacies should be involved in to support community outreach and community wellbeing?
13. Do you have any other comments you would like to add?

## Appendix B. Interview Questions for Commissioner, CCG and LPC

1. Please can you explain your role in the COVID champion service?
2. What feedback have you had from the pharmacies that have been involved?
3. I know only a small part, but please could you tell me about your involvement in the community outreach work
4. What would you want the legacy of the work to be?
5. Any other comments?

## Appendix C

**Table A1:** COREQ Checklist.

	Item No.	Guide Guides/Description	On Page No.
		Domain 1: Research team and reflexivity	
Interviewer/facilitator	1	Which author/s conducted the interview or focus group? RM	Methods—4
Credentials	2	What were the reseachers credentials? RM-PhD, MPharm	Title page
Occupation	3	What was their occupation at the time of the study? RM- Associate Professor	Methods—5
Gender	4	Was the researcher male of female? Female	Methods—4
Experience and training	5	What experience or training did the researcher have? RM –5 years of prior experience of qualitative research	Methods—4
		Relationship with participants	
Relationship established	6	Was a relationship established prior to the study commencement? Yes	Methods—4
Participant knowledge of the interviewer	7	What did the participants know about the researcher? e.g., personal goals, reasons for doing the research Participants were made aware about the evaluation of the service they were providing and the researcher credentials	Methods—4
Interviewer characteristics	8	What characteristics were reported about the interviewer/facilitator? e.g., Bias, assumptions, reasons and interests in the research topic PhD and pharmacist	Methods—4
		Domain 2: Study design	
		Theoretical framework	
Methodological orientation and Theory	9	What methodological orientation was stated to underpin the study? e.g., grounded theory, discourse analysis, ethnography, phenomenology, content analysis Content analysis	Methods—4
		Participant selection	
Sampling	10	How were the participants selected? e.g., purposive, convenience, consecutive, snowball Purposive	Methods—4
Method of approach	11	How were the participants approached? e.g., face-to-face, telephone, mail, email Telephone	Methods—4
Sample size	12	How many participants were approached? 20 pharmacists plus CCG, LPC and commissioner representative initially contacted.	Methods—4 Results—8
Non-participation	13	How many people refused to participate or dropped out? Reasons? 12 pharmacists gave contact details through a survey. All who gave details were interviewed.	Results—2
		Setting	
Setting of data collection	14	Where was the data collected? e.g., home, clinic, workplace Telephone	Methods—4
Presence of non-participants	15	Was anyone else present besides the participants and researchers? No other individuals were present	Methods—5
Description of sample	16	What are the important characteristic of the sample? e.g., demographic data, date Interviews were conducted between November-December 2021 15 interviews—12 pharmacists; 1 CCG, 1 LPC, 1 commissioner representative	Methods—4 Results—8
		Data collection	
Interview guide	17	Were questions, prompts, guides provided by the authors? Was it pilot tested? Semi structured interviews were used No pilot testing	Methods—3
Repeat interviews	18	Were repeat interviews carried out? If yes, how many? No	
Audio/visual recording	19	Did the research use audio or visual recording to collect the data? All interviews were audio recorded and transcribed	Methods—4
Field notes	20	Were field notes made during and/or/ after the interview or focus group? No additional notes were made	Methods—4
Duration	21	What was the duration of the interviews or focus groups? They lasted between 7–16 min	Methods—4
Data saturation	22	Was data saturation discussed? All those who agreed to participate were included	Methods—4
Transcripts returned	23	Were transcripts returned to participants for comments and/pr correction? No	
		Domain 3: analysis and findings	
		Data analysis	
Number of data coders	24	How many data coders coded the data? Transcripts were read by one member of the research team with availability for the rest of the research team (RM)	Methods—5
Description of the coding tree	25	Did authors provide a description of the coding tree? Questions were used as codes	Methods—5
Derivation of themes	26	Were themes identified in advance or derived from the data? As content analysis was used themes have been derived in advance by use of specific questions	Methods—5
Software	27	What software, if applicable, was used to manage the data? Data was analysed manually	Methods—5
Participant checking	28	Did participants provide feedback on the findings? No	
		Reporting	
Questions presented	29	Were participant quotations presented to illustrate the themes/findings? Was each quotation identified? e.g., participant number Comments were supported with direct quotes from participants who were anonymised	Methods—5 Results—8–10
Data and findings consistent	30	Was there consistency between the data presented and the findings? Yes	
Clarity of major themes	31	Were major themes clearly presented in the findings? Yes	
Clarity of minor themes	32	Is there a description of diverse cases or discussion of minor themes? No	
